# Pulse wave velocity in the aortic arch is the strongest predictor of left ventricular concentric remodeling in subjects with different levels of cardiovascular risk

**DOI:** 10.1186/1532-429X-11-S1-P13

**Published:** 2009-01-28

**Authors:** Alban Redheuil, Wen Chung Yu, Raymond T Yan, Elie Mousseaux, Alain De Cesare, David A Bluemke, Joao AC Lima

**Affiliations:** 1 Johns Hopkins University, Baltimore, MD USA; 2 INSERM U678, Paris, France; 3 NIH, Bethesda, MD USA

**Keywords:** Aortic Arch, Pulse Wave Velocity, Leave Ventricular Mass, Aortic Stiffness, Augmentation Index

## Introduction

Increased systolic blood pressure (SBP), pulse pressure (PP) and left ventricular (LV) concentric remodeling are associated with aging and increased cardiovascular risk. The aortic arch accounts for most of the vascular buffering function and is primarily involved in arterial stiffening and complications. Mechanisms of heart failure during aging and the role of aortic-ventricular stiffening and interaction need clarification. In particular, the importance of the aortic arch on central arterial stiffness remains unclear.

## Purpose

In this study, we evaluated the contribution of aortic arch stiffness to LV concentric remodeling

## Methods

We studied 52 subjects: 28 men; mean age: 41 yrs [20–79], 40 without and 12 with risk factors (hypertension in all and diabetes in 6) and normal LV ejection fraction. Global aortic stiffness was determined from carotid-femoral pulse wave velocity (cfPWV) using tonometry and transit surface distances. Aortic stiffness in the aortic arch was determined by MRI from as regional pulse wave velocity (PWV): ratio of aortic length to flow transit time. Augmentation index was calculated from tonometry as the ratio of the pressure above the inflection point and PP. SBP, MBP and PP were averages of 6 brachial measurements. End diastolic LV mass was measured on cine MRI. LV mass to volume ratio (M/V) was calculated as end diastolic volume over mass. Correlations are given and multivariate regression was used to study the determinants of concentric LV remodeling (M/V)

## Results

M/V ratio increased with age: r = 0.35 (p = 0.01); mean M/V in subjects <50 years is 0.97 vs. 1.18 in subjects >= 50 years (p = 0.006). Aortic arch PWV increased with age: r = 0.77 (p < 0.001), SBP: r = 0.62 (p < 0.001), PP: r = 0.69 (p < 0.001) and was well correlated with cfPWV: r = 0.83 (p < 0.001). However, PWV in the aortic arch was a stronger determinant of M/V ratio than global cfPWV in subjects >50 years. Moreover, in multivariate analysis using age, BMI, SBP, PP, augmentation index and cfPWV, PWV in the aortic arch was the strongest independent determinant of M/V ratio (R = 0.43; p < 0.001)

## Conclusion

Stiffness of the aortic arch directly measured by MRI as regional PWV was a stronger predictor of LV concentric remodeling than global cfPWV, age or blood pressure in a population sample with varied cardiovascular risk factors.

